# From Molecular Alterations to Risk Prediction: Biomarkers of Actinic Keratosis Progression to Cutaneous Squamous Cell Carcinoma

**DOI:** 10.3390/cancers18193221

**Published:** 2026-10-06

**Authors:** Thomas Meyer, Lea Stockfleth, Kerstin Lang, Heiko U. Käfferlein, Thomas Brüning, Eggert Stockfleth

**Affiliations:** 1 Department of Dermatology, St. Josef Hospital, Ruhr University Bochum, 44791 Bochum, Germany; l.stockfleth@web.de (L.S.); eggert.stockfleth@klinikum-bochum.de (E.S.); 2 Institute for Prevention and Occupational Medicine of the German Social Accident Insurance (IPA), Ruhr University Bochum, 44789 Bochum, Germany; kerstin.lang@dguv.de (K.L.); heiko.kaefferlein@dguv.de (H.U.K.); thomas.bruening@dguv.de (T.B.)

**Keywords:** skin cancer, malignant progression, biomarker, proliferation, differentiation, invasion, carcinogenesis

## Abstract

The high—and in most countries steadily increasing—incidence of cutaneous squamous cell carcinoma (cSCC) represents a growing public health challenge. As the majority of cSCCs arise from pre-existing actinic keratosis (AKs), these lesions are recognized as the principal precursors of invasive disease. A comprehensive understanding of the molecular mechanisms driving the progression from AK to cSCC is essential for advancing prevention, facilitating early diagnosis, and enabling targeted therapies. Moreover, this knowledge provides a basis for identifying biomarkers that can predict the risk of malignant transformation. The progression from AK to cSCC is a complex, multistep process driven by the gradual accumulation of genetic, epigenetic, and microenvironmental alterations. Over the past decades, numerous molecular changes associated with this transition have been identified. However, no single biomarker has demonstrated sufficient predictive accuracy for clinical use. This reflects the pronounced molecular heterogeneity of AKs as no individual alteration is uniformly present in all lesions that undergo malignant progression. In addition, numerous proposed biomarkers are also detected in non-progressive AKs and even in non-neoplastic skin, which compromises their sensitivity and specificity for predicting progression to cSCC. Current evidence, therefore, suggests that the most promising strategy for identifying AKs at greatest risk of malignant transformation is the development of a multi-marker panel. This panel, ideally, should integrate molecular signatures encompassing genetic, epigenetic, proliferative, inflammatory, and microenvironmental markers. This review summarizes the molecular factors that currently represent the most promising candidates for inclusion in such a multi-marker panel and discusses their potential clinical utility, while emphasizing the need for validation in well-designed prospective longitudinal studies.

## Simple Summary

Actinic keratosis (AK) is a precursor to skin cancer that, if left untreated, can develop into non-melanoma skin cancer—specifically, cutaneous squamous cell carcinoma (cSCC). However, progression to carcinoma occurs only in a small proportion of cases. To date, it is not possible to predict which specific AK lesion will develop into cSCC. Numerous factors associated with the malignant progression of AK have been described, including patient-specific, clinical, histological, and molecular factors. None of these factors serve as a standalone marker for predicting malignant progression, as the development of invasive cSCC is driven by the dysregulation of multiple, individually variable molecular signaling pathways rather than by alterations in a single signaling cascade. The use of multiple markers as a molecular signature could enable a more accurate assessment of progression risk in the future. This review presents the molecular factors that are primary candidates for this purpose.

## 1. Introduction

Cutaneous squamous cell carcinoma (cSCC) is the second most common malignant skin tumor after basal cell carcinoma and accounts for approximately 20% of non-melanoma skin cancers [1]. cSCC occurs predominantly in older adults; the average age at first diagnosis is around 70 years, and over 80% of cases affect individuals 60 years of age and older. Men are affected about twice as often as women [2,3].

The incidence of cSCC shows marked geographical variations. In the United States there are approximately 1.1 million new cases of cSCC annually. cSCC is not included in the national tumor registries, thus exact incidence data are difficult to collect. In a population-based study in Minnesota, the age-standardized incidence rates from 2000 to 2010 were adjusted to the population structure of the total United States and revealed incidence rates for males and females of 208 and 129 cases per 100,000 person-years, which is an overall increase in incidence of 263% compared to the time period between 1976–1984 [4].

In Europe, the overall incidence of cSCC is lower, but there are significant regional variations [5]. In all countries analyzed in that study the age-standardized incidence rates per 100,000 person-years were higher in men than in women. When comparing different time periods, the incidence rates for both men and women were consistently increasing [5]. In line with these trends a recently published large nationwide, population-based study from Denmark, based on data from the Danish Pathology Registry and Cancer Registry and comprising over 100,000 cases, showed that the age-standardized incidence rate of cSCC increased from 2005 to 2023. The estimated annual percentage change was 2.6% for males and 3.1% for females, reaching incidence rates of 131.6 and 77.7 per 100,000 person-years, respectively [6]. Furthermore, several European countries also show rising mortality trends, particularly in older populations [7].

In a recently published study from Germany, cancer registry data of four federal states were analyzed and showed that between 1986 and 2019, both crude and age standardized incidence rates increased by >500%. This increase was slightly greater in women than in men, with an average annual percentage change of 6.3% versus 5.7%. After peaking in 2015, incidence stabilized or declined slightly in three of the four states [8].

Australia exhibits the highest reported incidence of cSCC worldwide, with an overall rate of 467 cases per 100,000 person-years though being gender-specific too, i.e., 573 per 100,000 in men and 371 per 100,000 in women [9]. Despite these high levels, more recent analyses show a plateau or downward trend in incidence in Australia, while it continues to rise in most European countries and North America [10]. A study using the Global Burden of Disease (GBD) database from 2023 confirmed high prevalence of cSCC in high-income Western countries and showed a worldwide increase in cSCC case numbers between 1990 and 2023 [11].

The pronounced regional differences are primarily explained by variations in exposure to ultraviolet (UV) radiation, latitude, population aging, and the high proportion of fair skin types in highly exposed regions. Consequently, a clear epidemiological gradient exists, with the highest incidence rates observed in Australia, followed by North America, and lower, although steadily increasing, rates across Europe. Mortality is relatively low (approximately 1–2%) because cSCC metastasizes relatively rarely (approximately 4% will develop nodal metastases), and many tumors are therefore detected and treated early [12]. However, the global burden of disease caused by cSCC is likely to increase as indicated in the study using the 2023 GBD-database, predicting cSCC-related disability-adjusted life years (DALYs; years of life lost due to premature death plus years lived with disability) increase from 1.2 million in 2025 to 4.0 million in 2050 [11].

The high and, in most countries, rising incidence of cSCC, as well as increasing DALYs and mortality rates have significant implications for public health. Actinic keratoses (AK) represent the most important precursor to cSCC. Understanding the malignant transformation from AK to cSCC is therefore of key importance for prevention, early detection, and effective treatment of cSCC by providing the basis for development of biomarkers to predict the course of the disease, and characterization of potential target structures for therapeutic interventions [13].

## 2. Progression from AK to cSCC

AK are intraepidermal dysplasias of keratinocytes caused by chronic UV exposure. The incidence of AK correlates with cumulative UV exposure, as reflected by the higher frequency of AK in populations exposed to more intense solar radiation [13,14]. AKs are not merely regarded as premalignant lesions but are considered as early stages within the continuum leading to cSCC [15].

Histological and molecular similarities between AK and invasive cSCC, as well as the observation that the majority of cSCCs arise from pre-existing AKs, underscore their importance as direct precursor lesions [16]. Particularly in cases of multiple lesions, indicative of “field cancerization,” the overall risk of developing cSCC increases considerably [17]. The risk of malignant progression of individual AKs is generally low, but variable. Estimated annual progression rates per single lesion between 0.025–16% were described [18,19,20]. In a large prospective study, the risk of progression of a single AK lesion was 0.6% after one year and 2.6% after four years [21]. At the patient level the cumulative risk over several years is significantly higher and increases particularly with numerous AKs, advanced UV damage, or immunosuppression [13,19]. In individuals with multiple AKs, the cumulative lifetime risk of developing invasive squamous cell skin cancer is 6–10% [22].

On the other hand, a considerable number of AKs (25–60%) may regress spontaneously [18,20,23], however, they can also recur or develop anew in clinically unremarkable skin areas with about the same frequency, so that the absolute number does not change [18,23]. Due to this dynamic nature of AKs and their associated, albeit generally moderate, risk of progression, consistent therapy, regular dermatological check-ups, and effective sun protection are considered key measures for the prevention of cSCC. Furthermore, optimal management of AK and prevention of cSCC, require consideration of patient-related risk factors that are associated with lesion progression and the identification of molecular alterations that have been linked to AK progression. Despite the substantial burden of UV-induced mutations characteristic of all stages of this progression, it remains challenging to identify specific genomic alterations associated with the progression from AK to invasive cSCC, as both AK and cSCC exhibit similar mutations as well as chromosomal instability [24]. However, the expression levels of specific cancer-associated genes may vary during progression from normal skin, through AK, to invasive cSCC.

## 3. Patient-Related Risk Factors of AK Progression

Risk factors for progression of AK can be divided into patient-related and lesion-related factors. Patient-related factors are summarized in Table 1. The most significant risk factor for the development and progression of AK is chronic exposure to UV radiation. Cumulative UV exposure over many years leads to DNA damage in epidermal keratinocytes. This results in the emergence of characteristic mutations, including those in the tumor suppressor gene p53, which can lead to the clonal expansion of atypical cells. Accordingly, people in outdoor professions, such as construction workers, farmers, or gardeners, frequently exhibit pronounced chronic sun damage [25]. The same applies to individuals with intensive recreational exposure to sunlight or artificial UV radiation.

Older patients exhibit an increased risk of developing multiple AK lesions, as well as a higher likelihood of progression to invasive SCCs, as advanced age primarily reflects cumulative UV exposure and goes along with reduced capacities for DNA repair [26].

Another significant risk factor is a light skin type according to the Fitzpatrick classification (Type I–II). Patients with fair skin have less innate UV protection from melanin and are therefore more prone to chronic sun damage. Individuals with blonde or red hair, light-colored eyes, and a tendency to sunburn are particularly affected [27,28].

Immunosuppressed patients develop multiple AKs and invasive SCCs significantly more frequently than immunocompetent individuals [29]. In particular, organ transplant recipients are considered a high-risk group, as long-term immunosuppressive therapy impairs immunological control over atypical keratinocytes [30,31]. In addition, some immunosuppressive drugs have also been shown to promote tumor formation independent of their actions on the host immune system [32]. Consequently, premalignant cell clones may persist and more readily progress to invasive tumors. Moreover, SCCs in immunosuppressed patients often follow a more aggressive course and exhibit higher rates of recurrence and metastasis [19,33].

A history of previous cSCC or other forms of epithelial skin tumors also represents a strong risk marker. These patients frequently exhibit so-called field cancerization, that is, extensive areas of chronically UV-damaged skin containing numerous genetically altered keratinocytes [17,34]. The presence of multiple AKs is therefore regarded not only as a manifestation of chronic UV damage but also as an indicator of an increased risk of further malignant transformation.

Furthermore, male gender appears to be associated with a higher risk of progression. This is primarily attributed to greater cumulative UV exposure as well as occupational risk factors. Other potential influencing factors include genetic predispositions, such as DNA repair defects seen in xeroderma pigmentosum. Other genodermatoses, like epidermodysplasia verruciformis, and epidermolysis bullosa dystrophica, are also risk factors for cSCC development [35,36].

The role of human papillomaviruses (HPV) in the pathogenesis of cSCC is also being discussed, particularly in immunosuppressed patients [37,38]. Some studies suggest that certain HPV subtypes can promote carcinogenesis [39]. Smoking is likewise considered a potential additional risk factor, as oxidative stress and carcinogenic substances can facilitate tumor development [40].

In addition to classic patient-related risk factors, there are clinical signs that may indicate an increased likelihood of individual lesions progressing. These include persistent or treatment-resistant AKs, confluent plaques, increasing hyperkeratosis, induration, pain, bleeding, ulceration, or rapid growth of the lesion. Such changes should always prompt close monitoring or histological evaluation.

## 4. Histological Subtypes as Risk Factors for Progression

AKs are classified into various variants based on their microscopic (histological) appearance: hypertrophic AK with marked hyperkeratosis; atrophic AK with a thinned epidermis; acantholytic AK with suprabasal cleft formation; bowenoid AK with near-total epidermal atypia; pigmented AK with increased melanin deposition; and lichenoid AK with a dense lymphocytic infiltrate [41]. Bowenoid and hypertrophic AKs are often regarded as potentially more aggressive due to the pronounced cellular changes (atypia) observed histologically. However, convincing evidence that these histological subtypes alone confer a higher individual risk of progression to invasive squamous cell carcinoma is lacking [41]. Rather, all AKs are considered early precursors of non-melanoma skin cancer and have the potential to undergo malignant transformation.

Histologically, AKs can be classified into grades AK I, AK II, and AK III based on the proportion of the epidermis involved (atypical/dysplastic keratinocytes in the lower third, the lower two-thirds, or the entire thickness of the epidermis, respectively). As both AK and in situ SCC share the same clinical, histological, and molecular features, AKs are usually considered as early in situ SCC rather than pre-malignant neoplasms; consequently, it has been recommended to use the terminology “early in situ SCC type AK I”, “early in situ SCC type AK II”, and “in situ SCC type AK III” [41]. However, the development of invasive cSCC does not always follow a stepwise progression from AK I via AK II to AK III—the classic pathway of transformation. Most AKs associated with invasive cSCC correspond to histological grade AK I and indicate direct invasion originating from the basal cell layer (differentiated pathway) [42].

Dermal invasion by tumor cells originating in the epidermis requires crossing of the basal membrane. Accordingly, classification based on the spread pattern of atypical basal keratinocytes has proven prognostically relevant. Three proliferation patterns are distinguished. In PRO I (crowding), densely packed atypical basal keratinocytes create a basophilic appearance without architectural distortion. In PRO II (budding), small hemispherical nests of atypical keratinocytes protrude into the papillary dermis, remaining thinner than the overlying epidermis. PRO III (papillary sprouting) is characterized by spiky or filiform projections of atypical keratinocytes extending into the upper dermis, exceeding the thickness of the epidermis above [43].

Compared to the clinical classification of AK according to Olsen or the AK I–AK III grading system, the extent of basal proliferation correlates significantly better with the presence of invasive cSCC [44,45]. Although the PRO score is a strong histological indicator, the exact timing and precise risk of transformation for an individual lesion remains difficult to predict.

Reliable prediction for individual lesions is currently impossible, neither clinically nor histologically. At present, there is insufficient evidence that histomorphological parameters may translate into clinically and/or therapeutically relevant consequences.

## 5. Molecular Changes Associated with AK Progression

The progression of AK to cSCC is a complex, multistep process involving various molecular alterations. The key molecular factors and signaling pathways identified in this context are listed in Table 2. They mostly represent markers of carcinogenesis and biological aggressiveness, but not necessarily predictors of future transformation. The predictive value of individual markers regarding the risk of progression is generally limited. Therefore, combining multiple molecular alterations with patient-specific clinical and histological factors could significantly improve the prediction of malignant progression.

### 5.1. TP53/p53 Family

UV-induced *TP53* mutations represent the most common genetic alterations in cSCC and are considered early events in tumorigenesis [46]. Characteristic features include UV-signature mutations, specifically C→T and CC→TT transitions, which are detectable even in AK [47,48]. These mutations promote the clonal expansion of atypical keratinocytes and contribute to further genetic instability, thereby facilitating additional alterations in tumor suppressor genes and oncogenes. The prevalence of *TP53* mutations is approximately 50–60% in primary cSCC, and in up to 95% of cases of metastatic cSCC. However, *TP53* mutations are found not only in invasive tumors but also in chronically UV-exposed, clinically normal-appearing skin, in AK, and in carcinoma in situ [47,49]. *TP53* mutations have been detected significantly more frequently in sun-exposed skin than in non-exposed skin (approx. 74% vs. 5%) [50].

These findings demonstrate that *TP53* mutations occur very early during UV-induced carcinogenesis and form the basis for the accumulation of further driver mutations. Though *TP53* mutations constitute a key molecular feature of cSCC, their utility as a standalone marker of disease progression is limited as they are also detected in a high proportion of AKs. It is also crucial to note that the consequences of *TP53* mutations for p53 protein expression are heterogeneous and depend on the underlying mutation type. Missense mutations typically produce a stable full-length mutant protein with prolonged half-life, resulting in diffuse nuclear accumulation, whereas nonsense or frameshift (null) mutations usually lead to complete loss of detectable p53 expression. Truncating mutations may show variable immunohistochemical detectability [51]. Accordingly, immunohistochemical analyses yield inconsistent results; however, many studies have demonstrated increased p53 expression in atypical keratinocytes of AK. In most cases, this increased immunoreactivity reflects the accumulation of a dysfunctional mutant p53 protein with an extended half-life, making p53 immunohistochemistry a useful surrogate marker for underlying *TP53* mutations [52]. Nevertheless, the relationship between specific *TP53* mutations, p53 protein stability, and functional activity remains incompletely understood, and further mechanistic studies are required to elucidate how individual mutations influence p53 structure, stability, and biological function [53].

Besides the early acquisition of UV-induced *TP53* mutations, alterations in *TP63* expression and dysregulation of the p38 mitogen-activated protein kinase (MAPK) pathway contribute to the progression of AK towards invasive cSCC. Although neither pathway appears to initiate carcinogenesis independently, both influence the balance between keratinocyte proliferation, differentiation, stress adaptation and invasion, thereby facilitating malignant progression.

*TP63*, a member of the *TP53* gene family, is crucial for epidermal development and the maintenance of epithelial stem cell populations [54]. In adult skin, the predominant ΔNp63 isoform is expressed in basal keratinocytes, where it preserves proliferative capacity while preventing terminal differentiation [55]. In AK and cSCC, ΔNp63 expression is frequently increased and extends beyond the basal layer, reflecting expansion of immature keratinocyte populations during neoplastic progression [56]. Functionally ΔNp63 contributes to sustaining the proliferation of UV-damaged keratinocytes and antagonizes p53-dependent apoptotic programs, promoting an undifferentiated, proliferative cell phenotype, whereas UV-induced p38 activation promotes ΔNp63 degradation, thereby enabling p53-mediated elimination of damaged cells [57]. Although TP63 overexpression is consistently observed during cSCC development, and in some cases, a more aggressive tumor type, it has not been shown that TP63 alone reliably predicts the risk of progression for an individual AK lesion [58].

The p38-MAPK pathway functions as a central sensor of cellular stress and mediates cellular responses to UV radiation, oxidative stress, DNA damage, and proinflammatory cytokines [59]. Its role during cutaneous carcinogenesis is highly context dependent. Following acute UV exposure, p38 acts predominately as a tumor suppressor by activating cell-cycle arrest, DNA repair and apoptosis thereby facilitating the elimination of genetically damaged keratinocytes. However, in chronically UV-exposed skin, persistent activation of p38 can contribute to tumor progression by enhancing the production of pro-inflammatory cytokines and matrix-remodeling enzymes, thereby promoting extracellular matrix degradation, cellular migration and tissue invasion [60,61].

Overall, the biological functions of both signaling pathways complement each other in the model of cSCC development. Whereas ΔNp63 maintains the proliferative and regenerative capacity of genetically altered keratinocytes, chronic p38 activation promotes inflammatory signaling, extracellular matrix remodeling and invasion. Together with early UV-induced *TP53* mutations and other genetic alterations, such as those in *NOTCH1*, *CDKN2A* or *hTERT* promoter, these molecular changes contribute significantly to the progression from AK to invasive cSCC.

### 5.2. Notch Signaling Pathway

The Notch signaling pathway is a key regulator of epidermal homeostasis and functions predominantly as a tumor suppressor during cutaneous squamous carcinogenesis. In contrast to many other malignancies, in which aberrant Notch activation exerts oncogenic effects, loss of Notch signaling represents a hallmark of the development of cSCC [62].

In normal skin, Notch signaling promotes the transition of basal keratinocytes from proliferation to terminal differentiation, thereby maintaining epidermal architecture and limiting the expansion of progenitor cell populations [62,63]. Chronic UV radiation frequently induces loss-of-function mutations in *NOTCH1* and *NOTCH2*, which are among the earliest and most common genetic alterations detected in chronically sun-exposed skin, AK and invasive cSCC [49]. These mutations often coexist with UV-induced *TP53* alterations, indicating that impairment of both tumor suppressor pathways occur during the earliest stages of field cancerization. Diminished Notch signaling impairs keratinocyte differentiation, prevents timely cell-cycle exit and promotes the persistence and expansion of progenitor-like cell populations, thereby disrupting epidermal homeostasis [64].

Beyond its role in regulating differentiation, Notch signaling suppresses keratinocyte proliferation by functionally antagonizing multiple mitogenic pathways, including EGFR-, Ras/MAPK-, PI3K/AKT-, and Wnt/ß-catenin-dependent signaling [65]. Crosstalk between Notch and p53 further reinforces this tumor suppressive network, as wild-type p53 directly activates *NOTCH1* transcription. Consequently, loss of p53 function can attenuate Notch signaling, thereby further impairing epidermal differentiation and promoting cSCC development [66].

Emerging evidence also indicates that Notch signaling influences the tumor microenvironment. Reduced Notch activity is associated with enhanced activation of NF-κB, increased production of pro-inflammatory cytokines such as IL-6 and IL-8, and recruitment of inflammatory immune cells, thereby establishing a tumor-promoting inflammatory microenvironment [62]. Furthermore, loss of Notch signaling promotes extracellular matrix remodeling, impairs epithelial cell adhesion, and facilitates keratinocyte migration and local tissue invasion, in part through increased matrix metalloproteinase expression and dysregulation of adhesion molecules including E-cadherin, thereby supporting progression from intraepidermal neoplasia to invasive cSCC [62,64].

These findings therefore support a model in which attenuation of Notch signaling does not independently trigger malignant transformation but lowers the threshold for tumor growth by impairing keratinocyte differentiation, promoting their sustained proliferation, and facilitating the accumulation of further oncogenic alterations. Together with *TP53* inactivation and the dysregulation of signaling pathways such as EGFR, p38 MAPK, PI3K/AKT and Wnt/ß-catenin, the loss of Notch signaling constitutes a key molecular event driving the progression from AK to invasive cSCC.

### 5.3. CDKN2A (p16INK4a, p14ARF)

*CDKN2A* encodes two alternatively spliced tumor suppressor proteins, p16INK4a und p14ARF, which inhibit cell-cycle progression and proliferation through the RB and *TP53* pathways, respectively [67]. In normal epidermis, activation of *CDKN2A* (particularly p16INK4A) in response to UV-induced DNA damage promotes cell-cycle arrest and senescence of damaged keratinocytes, thereby limiting the expansion of genetically unstable cell clones [68].

In AK, p16INK4a is frequently overexpressed, reflecting activation of an oncogene- and DNA damage-induced senescence program in response to chronic UV exposure rather than functional loss of *CDKN2A* or malignant transformation [69]. This senescence response is considered an important barrier that restrains the expansion of genetically altered keratinocyte clones. Consequently, p16 expression is often higher in AKs than in normal epidermis, though this does not necessarily predict progression to invasive carcinoma.

Progression from AK to invasive cSCC is associated with functional escape from this tumor suppressive barrier. Deletions, mutations, or epigenetic alterations of *CDKN2A* occur more frequently in invasive cSCC than in AK, resulting in a loss of cell cycle control [70,71]. Although *CDKN2A* alterations are detected in a subset of cSCC, they occur considerably less frequently than *TP53* and *NOTCH1/2* mutations and are considered secondary events acquired during tumor progression rather than initiating drivers [72]. Moreover, disruption of the RB or p53 pathways can abrogate *CDKN2A*-mediated tumor suppression even in the presence of persistent p16 expression. In such cases, p16 immunoreactivity does not indicate preserved tumor suppressor activity but reflects a compensatory response to deregulated RB signaling or UV-induced cellular senescence. Thus, p16 expression alone has not consistently been shown to predict malignant progression to invasive cSCC [73,74].

Overall, current evidence supports a predominantly tumor suppressive role for *CDKN2A* during the early phases of keratinocyte carcinogenesis through the induction of cell cycle arrest and senescence. However, progression to invasive cSCC appears to be driven by the acquisition of additional genetic alterations that overcome *CDKN2A*-dependent cell-cycle control and senescence. Consequently, p16 expression is not currently considered as a reliable biomarker for predicting the progression of individual AK lesions to invasive cSCC. On the other hand, *CDKN2A* alterations, although not among the earliest or most frequent mutations in the carcinogenesis of squamous cell carcinoma, correlate with adverse clinical outcomes, such as greater tumor extent and deeper invasion [71]. Since *CDKN2A* mutations are associated with disease-specific death in metastatic cSCC, they may represent a potential prognostic biomarker for disease-specific survival, although validation in larger independent cohorts is still required [71].

### 5.4. EGFR (Epidermal Growth Factor Receptor)

While EGFR signaling is essential for normal epidermal homeostasis and wound repair [75], chronic UV radiation induces persistent EGFR activation, promoting the proliferation, survival and clonal expansion of UV-damaged keratinocytes through activation of the MAPK, PI3K–AKT–mTOR and JAK–STAT signaling pathways [76,77].

Under physiological conditions, activated EGFR is rapidly internalized and degraded, thereby preventing prolonged receptor signaling [78]. During AK development, EGFR signaling undergoes progressive dysregulation, characterized by increased EGFR expression, release of EGFR ligands such as transforming growth factor-α (TGF-α), amphiregulin (AREG) and heparin-binding EGF-like growth factor (HB-EGF), as well as amplified downstream MAPK and PI3K–AKT signaling [76,79]. These changes promote keratinocyte proliferation, impair terminal differentiation, increase resistance to UV-induced apoptosis and facilitate the clonal expansion of UV-damaged keratinocytes.

In invasive cSCC, EGFR levels are further elevated, and the receptor is constitutively activated, leading to persistent tumor cell proliferation, enhanced survival, and resistance to apoptosis [80]. Unlike in several other epithelial malignancies, EGFR signaling in cSCC is only rarely driven by activating EGFR mutations. Instead, aberrant EGFR activity primarily results from receptor overexpression, increased autocrine production of EGFR ligands, and extensive crosstalk with other receptor tyrosine kinases and downstream oncogenic signaling pathways, including MAPK and PI3K–AKT [80].

Although EGFR activity is increased in many AKs, numerous lesions remain clinically stable or even regress, particularly in the absence of additional alterations affecting key tumor suppressor pathways such as *TP53*, *NOTCH1* and *CDKN2A* [13]. Consequently, EGFR activation alone appears to have limited value as a biomarker for predicting AK progression [81].

The transition from intraepidermal neoplasia to invasive cSCC is thought to require cooperative interactions between sustained EGFR signaling and the loss of tumor suppressor pathways, particularly *TP53* and NOTCH signaling, which together promote proliferation, impair differentiation, and facilitate invasion [49,80]. Furthermore, EGFR signaling induces the expression of matrix metalloproteinases (particularly MMP1, MMP2, and MMP9) and transcription factors associated with epithelial-mesenchymal transition (EMT), including SNAIL, SLUG, TWIST, and ZEB1 [80,82]. Collectively, these observations suggest that downstream mediators of EGFR signaling, including MMPs and EMT-associated transcription factors, may provide more informative biomarkers of AK progression than EGFR expression alone, although prospective validation is still required [80,82,83].

### 5.5. Matrix-Metalloproteinases (MMPs)

The transition from AK to invasive cSCC requires the breakdown of the basement membrane and the extracellular matrix. This function is mediated by MMPs, in particular MMP-1, MMP2, MMP-9, and MMP-13 [84]. In normal epidermis, MMP expressions are low and tightly regulated. Chronic UV exposure activates EGFR signaling and its downstream MAPK–AP-1 and NF-κB pathways, resulting in the transcriptional induction of multiple matrix metalloproteinases, particularly MMP1, MMP2 and MMP9 [80,84].

As AKs progress towards high-grade dysplasia, sustained EGFR signaling, chronic inflammation and tumor–stroma interactions lead to progressively increased MMP expression [85]. While this response contributes to photoaging and tissue remodeling, it is generally insufficient to promote invasion as long as tumor suppressor pathways such as *TP53*, NOTCH and *CDKN2A* remain intact [86]. MMPs are not regarded as initiating drivers of malignant transformation. Rather, their increased expression and activation represent downstream consequences of oncogenic signaling networks, including EGFR, MAPK and inflammatory pathways, through which MMPs remodel the extracellular matrix and tumor microenvironment to promote invasion and tumor progression [87,88].

Beyond matrix degradation, MMPs promote tumor progression by releasing matrix-bound growth factors, including transforming growth factor-β (TGF-β), vascular endothelial growth factor (VEGF) and EGFR ligands [87]. In addition, MMPs contribute to EMT-associated phenotypic plasticity by cleaving E-cadherin and other adhesion molecules, thereby weakening epithelial integrity, while simultaneously activating signaling cascades that induce EMT transcription factors such as SNAIL, SLUG, TWIST and ZEB1/2 resulting in increased cellular motility and invasiveness [87,89].

Consistent with these functional roles, several studies have demonstrated a stepwise increase of MMP expressions from normal skin to AK and finally to invasive cSCC, with MMP2, MMP9 and MMP14 showing the strongest associations with invasive growth and tumor aggressiveness [85,90,91]. However, elevated MMP expressions alone are insufficient to predict malignant progression of individual AK lesions, as MMPs are also induced by chronic UV exposure, wound repair and cutaneous inflammation [91,92]. Consequently, although MMPs represent robust markers of extracellular matrix remodeling and invasive potential, they lack the specificity required for use as standalone biomarkers for AK progression.

### 5.6. Epithelial—Mesenchymal Transition (EMT)

EMT has emerged as a key biological process during the progression of AK to invasive cSCC, promoting the acquisition of an invasive phenotype through the loss of epithelial cell–cell adhesion, and the gain of mesenchymal characteristics associated with enhanced cellular motility and invasiveness [24]. During this transition, keratinocytes undergo a partial rather than a complete EMT program, characterized by reduced E-cadherin-mediated cell–cell adhesion, induction of the EMT-associated transcription factors SNAIL, SLUG, TWIST1 and ZEB1/2, cytoskeletal remodeling with increased vimentin expression, and enhanced production of MMPs [82,93,94].

Bakshi et al. demonstrated a progressive reduction in E-cadherin expression from non-sun-exposed skin to chronically UV-exposed skin and subsequently to AKs, whereas no further decrease was observed between AKs and non-melanoma skin cancers [23]. These findings suggest that E-cadherin loss is primarily involved in the transition from normal epidermis to UV-damaged skin and the development of AK, rather than in the subsequent progression to invasive carcinoma. Consistent with this concept, the EMT-associated transcription factors SNAIL, SLUG and TWIST were significantly upregulated in UV-exposed skin and AKs compared with non-UV-exposed skin. Their increased expression, together with reduced E-cadherin levels, supports the notion that activation of EMT-associated transcriptional programs contributes to UV-induced epithelial remodeling and promotes the early stages of cutaneous carcinogenesis [23].

In addition to canonical EMT-inducing transcription factors, the Ovo-like zinc finger transcription factors OVOL1 and OVOL2 function as important suppressors of EMT by antagonizing ZEB1 expression [95]. Experimental inhibition of OVOL2 increases ZEB1 expression and enhances the invasive capacity of cSCC cells, highlighting the functional importance of this regulatory axis. Immunohistochemical analyses further demonstrated that OVOL1 and OVOL2 expressions are increased in AK but significantly reduced in cSCC, whereas ZEB1 expression is markedly elevated in invasive tumors. This reciprocal expression pattern suggests that disruption of the OVOL1/2–ZEB1 regulatory network may represent a critical molecular event during the transition from intraepidermal neoplasia to invasive cSCC [95].

Despite their central role in tumor invasion, current evidence does not support the use of individual EMT-associated factors as reliable predictors of malignant progression in AK. Many EMT-associated molecules are induced not only during malignant progression but also by chronic UV radiation, cutaneous inflammation and physiological wound repair, limiting their specificity as biomarkers for predicting progression of individual AKs [23,96]. Furthermore, partial EMT programs can be detected in lesions that remain clinically stable or regress spontaneously, whereas invasive cSCC may arise through heterogeneous invasion programs and does not invariably exhibit strong expression of classical EMT markers during the preceding intraepidermal stage [93]. Consequently, the expression of EMT-associated molecules alone lacks the sensitivity and specificity required for clinical risk stratification.

Instead, EMT should be regarded as one component of a complex molecular network governing malignant progression. Increasing evidence suggests that EMT does not act in isolation but cooperates with UV-induced genomic alterations, including *TP53* mutation and attenuation of NOTCH signaling, as well as persistent growth factor signaling, stromal remodeling and chronic inflammation to facilitate basement membrane invasion during progression from AK to invasive cSCC [72,80].

### 5.7. Other Potential Markers of AK Progression

Enhancer of Zeste Homolog 2 (EZH2): EZH2, the catalytic subunit of the Polycomb Repressive Complex 2 (PRC2), has emerged as an important epigenetic regulator of cutaneous squamous cell carcinogenesis. Through catalyzing trimethylation of histone H3 lysine 27 (H3K27me3), EZH2 represses differentiation-associated and tumor suppressor genes, thereby maintaining keratinocytes in a proliferative, undifferentiated state [97]. In addition, EZH2 contributes to epithelial–mesenchymal plasticity by repressing epithelial lineage genes and functionally cooperating with EMT-associated transcription factors including SNAIL, TWIST and ZEB [97,98]. EZH2 expression increases progressively from normal skin to AK and invasive cSCC, supporting a role for EZH2 in the stepwise development of cSCC [99,100]. More recently, Valea et al. [101] demonstrated that EZH2 is preferentially upregulated in high-risk cSCCs arising at the lip and ear and is associated with adverse clinicopathological features, including poor tumor differentiation and advanced American Joint Committee on Cancer (AJCC) stage, suggesting that EZH2 serves as a marker of aggressive tumor biology. Although no significant association with lymph node metastasis was observed, these findings further support the concept that EZH2 contributes to malignant progression and may help to identify biologically aggressive precursor lesions. Nevertheless, the prognostic value of EZH2 for predicting the clinical fate of individual AK lesions remains unproven. Current evidence therefore supports EZH2 primarily as a marker of biological aggressiveness rather than a validated standalone predictor of malignant transformation.

Cyclo-oxygenase-2 (COX-2): UV radiation induces COX-2 expression in keratinocytes through activation of MAPK signaling and the downstream transcription factors NF-κB and AP-1, leading to sustained prostaglandin E_2_ (PGE_2_) production in chronically sun-exposed skin. PGE_2_ promotes keratinocyte proliferation, inhibits apoptosis, stimulates angiogenesis and suppresses anti-tumor immune responses, thereby contributing predominantly to tumor promotion and progression rather than to tumor initiation [102,103]. COX-2 expression is detectable in AK and is generally further increased in invasive cSCC, consistent with a role for COX-2 in the progression of keratinocyte carcinogenesis [102]. Although COX-2 expression increases during progression from AK to cSCC, its clinical utility as a biomarker for predicting malignant transformation of individual AK lesions remains limited. COX-2 is an inducible inflammatory enzyme that is also upregulated during wound healing and cutaneous inflammation, resulting in substantial overlap between biologically indolent and high-risk lesions [104].

Human telomerase reverse transcriptase (hTERT): hTERT, the catalytic subunit of telomerase, contributes to cSCC development by preventing telomere shortening and enabling replicative immortality of UV-damaged keratinocytes [105]. Beyond telomere maintenance, hTERT promotes proliferation, suppresses senescence and apoptosis, enhances DNA damage tolerance and cooperates with oncogenic signaling pathways including WNT/β-catenin and NF-κB [106]. hTERT expression and telomerase activity are low or absent in normal epidermis, become detectable in AK and generally increase further in invasive cSCC, supporting the concept that telomerase activation contributes predominantly to malignant progression and cellular immortalization rather than representing an initiating event in cutaneous squamous carcinogenesis [105,107]. Unlike hTERT protein overexpression, which generally increases during progression from AK to invasive cSCC, *hTERT* promoter mutations represent an early genetic event and are already detectable in AK [108]. These mutations (predominantly C228T and C250T) generate de novo E26 transformation-specific (ETS) transcription factor binding sites, leading to transcriptional activation of *hTERT*, telomerase reactivation and facilitating the clonal expansion of UV-damaged keratinocytes [109]. However, the clinical utility of both hTERT protein expression and promoter mutations as a standalone biomarker for predicting malignant transformation of individual AK lesions remains uncertain because considerable overlap exists between non-progressive and progressive AKs [110], and prospective longitudinal studies for both hTERT protein expression and promoter mutations are lacking.

RAS: Activation of the RAS signaling pathway may also contribute to the progression of AK towards invasive cSCC. Ras molecules are a family of GTP-binding proteins including *HRAS*, *KRAS* and *NRAS* that are among the most frequently mutated genes in human cancers [111]. Ras is an upstream activator of the Raf/Mek/Erk1/Erk2 kinase pathway, and activating mutations in Ras can promote cSCC formation [111]. In contrast to *TP53* or *NOTCH1* mutations, activating RAS mutations are relatively rare in AK and cSCC, although activation of the RAS–RAF–MEK–ERK (MAPK) signaling pathway is frequently detectable [72,112,113]. Thus, functional activation of the RAS/MAPK signaling pathway is likely more important for the progression from AK to invasive cSCC than the presence of classic RAS mutations. RAS primarily functions as a progression-promoting event rather than an initiating driver, cooperating with pre-existing UV-induced genetic alterations, particularly *TP53* and *NOTCH1* mutations, to enhance keratinocyte proliferation, invasion, and tumor growth [114]. However, based on current evidence, activating RAS mutations are too infrequent and lack sufficient prognostic value to serve as a reliable predictive biomarker for the malignant transformation of individual AK [112].

Src-family tyrosine kinases (SFKs): SFKs and their negative regulator, Src-activating and signaling molecule (Srcasm), play opposing roles in the progression from AK to invasive cSCC. The Src family comprises non-receptor tyrosine kinases including Src, Fyn, Yes, Lyn, Lck, Hck, Fgr, and Blk, which regulate numerous cellular processes such as proliferation, differentiation, migration, and survival [115]. While SFKs promote proliferative and invasive signaling pathways, Srcasm acts as a tumor-suppressive antagonist whose loss facilitates malignant progression [49,116]. Activation of SFKs occurs predominantly as a secondary event driven by EGFR overexpression, integrin signaling, or the loss of negative regulators. In this sense SFKs act as central signal integrators that couple EGFR, integrin and inflammatory signaling pathways, thereby promoting keratinocyte proliferation, survival, migration and invasion [117]. In addition, progressive loss of Srcasm expression from normal epidermis through AK to invasive cSCC is associated with increased SFK activity and is thought to facilitate persistent SFK signaling during tumor progression [118]. Although increasing SFK activity and reduced Srcasm expression are associated with progression to from AK to cSCC, neither marker has been clinically validated as a reliable predictor of malignant transformation in individual AK lesions, largely because prospective longitudinal studies and validated diagnostic cut-offs are lacking.

KNSTRN: The *KNSTRN* gene has emerged as a significant player in the development and progression of AK and cSCC. Mutations in this gene, particularly the recurrent somatic mutations, have been linked to the acceleration of cSCC development. The *KNSTRN* gene encodes a protein that binds to Astrin on the kinetochore in the late stage of cell mitosis and is essential for proper chromosome alignment and segregation during cell division [119]. UV-induced mutations lead to disrupted chromosome segregation, aneuploidy, and chromosomal instability, which represent hallmarks of tumor progression [49]. Exome sequencing revealed somatic *KNSTRN* mutations in 19% of cSCCs from 112 patients; notably, the most common mutation, p.Ser24Phe, exhibited a characteristic UV-induced C > T mutation pattern [120]. A study comparing healthy skin tissue, chronically sun-damaged skin, various AK subtypes, and invasive cSCCs found *KNSTRN* mutations significantly more frequently in basally proliferating AKs (PRO II/III) and invasive cSCCs than in healthy or merely sun-damaged skin. In contrast, these mutations were markedly less common in AKs with low proliferative activity (PRO I) [121]. These findings suggest that *KNSTRN* mutations are associated with more invasive growth behavior and advanced tumor progression and may specifically promote the basally proliferating AK phenotype. Whether KNSTRN is suitable as a biomarker for predicting the malignant transformation of individual AKs remains unclear and requires investigation in prospective long-term studies.

β-Catenin: β-Catenin, encoded by the *CTNNB1* gene, is the central intracellular effector of the canonical Wnt signaling pathway and plays a pivotal role in regulating keratinocyte proliferation, differentiation, and epidermal tissue homeostasis [122]. While UV-induced *TP53* and *NOTCH1* mutations are among the earliest genetic events in the development of AK, activation of the Wnt/β-catenin signaling pathway appears to contribute primarily to tumor progression rather than tumor initiation [122]. Activating *CTNNB1* mutations are rare in human AK and cSCC [113]. Instead, the pathway is typically activated through alterations in regulatory components or through extensive crosstalk with other oncogenic pathways, including EGFR, PI3K/AKT, TGF-β, and Src signaling [122,123]. During the progression from AK to invasive cSCC, β-catenin progressively loses its membranous localization and shows increasing aberrant cytoplasmic and nuclear localization [122,124]. Following translocation into the nucleus, β-catenin interacts with TCF/LEF transcription factors and activates transcription of multiple target genes, including *CCND1* (Cyclin D1), *MYC*, and *BIRC5* (Survivin), thereby promoting cell proliferation and enhancing the survival of transformed cells [125]. Furthermore, enhanced β-Catenin signaling contributes to EMT, increases cell migration, extracellular matrix degradation, and invasive growth [94]. Experimental studies indicate that activation of the Wnt/β-catenin signaling pathway promotes cancer stem cell properties, thereby enhancing tumor growth, cellular plasticity, and resistance to anticancer therapies [122]. Although aberrant β-catenin signaling is associated with cSCC progression, significant inter-lesional variability limits its utility as a predictive biomarker for the malignant transformation of individual AK lesions [122].

Epigenetic factors: Epigenetic alterations are further characteristic features of malignant cell transformation and, across all forms of cancer, they are approximately ten times more frequent than mutations [126]. The regulation of gene expression by epigenetic factors involves numerous mechanisms, such as DNA methylation, histone modifications, chromatin remodeling, and regulatory RNAs. Unlike genetic mutations that alter the DNA sequence, epigenetic changes such as DNA methylation result in altered gene expression without changing the nucleotide sequence.

Aberrant DNA methylation is associated with the development and progression of cSCC. Alterations in DNA methylation are detectable already in AK and accumulate during progression to invasive cSCC, indicating that epigenetic dysregulation is an early and progressive event in cSCC development [127,128]. In particular, hypermethylation of promoter regions leads to epigenetic inactivation of tumor suppressor genes. Affected genes include those regulating cell cycle control, DNA repair, differentiation, and apoptosis. For instance, *CDKN2A* promoter methylation, which suppresses the expression of the tumor suppressors p14ARF and p16INK4A, is found in 40% of cSCC cases [129]. E-cadherin expression is also reduced via promoter hypermethylation during cSCC tumorigenesis. Chiles et al. detected promoter hypermethylation of the E-cadherin gene in 85% of invasive cSCCs, 50% of SCCs in situ, 44% of AKs, and 22% of non-neoplastic skin samples [130]. Although AKs and cSCCs exhibit similar epigenetic changes, the extent of DNA methylation increases significantly with tumor progression, suggesting that aberrant DNA methylation promotes the progression of carcinogenesis.

Non-coding regulatory RNAs mediate other mechanisms of epigenetic regulation involved in SCC development. These included micro RNAs (miRNA), long non-coding RNAs (lncRNA) and circular RNAs (circRNA) [131]. Among them, miRNAs have been studied in most detail to date. Mature miRNAs are short endogenous RNA molecules (17–23 nucleotides in length) that are involved in the post-transcriptional regulation of gene expression, by reducing or even fully blocking the translation of specific messenger RNAs (mRNA) [132]. They act either as tumor suppressors or as oncomirs (oncogenic miRNAs) and influence key processes such as cell proliferation, differentiation, apoptosis, EMT, invasion, and interaction with the tumor microenvironment.

The oncomir-1 cluster of six miRNAs (miR-17, miR-18a, miR-19a, miR-20a, miR-19b-1 and miR-92a-1), also known as mir-17–92, was the first described set of mammalian miRNA oncogenes. The oncomir-1 products regulate the expression of genes involved in cell proliferation, apoptosis and angiogenesis [133]. miR-17/92 was initially found amplified in hematopoietic malignancies, particularly B-cell lymphomas [133], but was also found to be significantly increased in cSCC [134]. miR-21 is another well-studied miRNA whose expression may already be elevated in AK and increases further in cSCC. miR-21 inhibits tumor suppressors such as PTEN and PDCD4, thereby activating PI3K/AKT signaling, reducing apoptosis, and promoting cell proliferation and invasion [135,136]. miR-31 is also significantly upregulated in invasive cSCC and is associated with increased MMP-1 expression [137]. Both factors were elevated particularly in well-differentiated, invasively growing tumors. At the same time, the authors report enhanced EMT, suggesting that miR-31 supports cSCC invasion by inducing MMP-1 and promoting partial EMT, thereby enabling tumor cells to develop a pronounced invasive capacity despite largely preserved differentiation [137].

In contrast, various tumor-suppressive miRNAs are downregulated in cSCC. For example, miRNA-34a, a downstream effector of p53 that targets and blocks several major cancer-promoting genes, including *MYC*, *NOTCH*, and *BCL-2* [138], is downregulated in cSCC, probably due to the frequent loss of *TP53* function resulting from UV-induced mutations [139]. miR-203, a key regulator of keratinocyte differentiation, is also frequently downregulated during progression from AK to cSCC and acts as a tumor suppressor by repressing the p63 transcription factor required for stem-cell maintenance in stratified epithelial tissues and upregulated in SCC [56,140]. In contrast, though miR-205 stabilizes epithelial identity and suppresses complete EMT through inhibition of ZEB transcription factors [141], its expression in cSCC is context-dependent and may even increase in invasive tumor regions despite its canonical anti-EMT function [142]. Regardless of their biological significance, no individual miRNA has yet been established as clinically validated biomarker that reliably predicts the malignant transformation of an individual AK.

## 6. Conclusions: Which Factors Are Best Suited to Predict the Malignant Progression of AK?

The development of cSCC is a multistep process driven by chronic UV exposure, resulting in the progressive accumulation of genetic and epigenetic alterations that promote the transition from AK to invasive carcinoma. The pathogenetic mechanisms involved in the development of cSCC have been described in several excellent review articles [35,49,86,143,144]. In brief: the earliest and most critical molecular step is UV-induced DNA damage, leading to characteristic mutations, particularly in the *TP53* gene. Loss of p53 function impairs DNA repair, cell cycle control, and apoptosis, allowing genetically damaged keratinocytes to survive and undergo clonal expansion. Subsequently, additional alterations in tumor suppressor genes such as *NOTCH1/2* and *CDKN2A* occur, disrupting epidermal differentiation and further promoting uncontrolled cell proliferation. Concurrently, pro-proliferative signaling pathways, such as EGFR, RAS/MAPK, and PI3K/AKT/mTOR, are activated, supporting tumor cell growth, survival, and metabolic adaptation. Furthermore, epigenetic changes, including aberrant DNA methylation and altered expression of regulatory miRNAs, contribute to the sustained dysregulation of numerous genes and progressively increase during tumor evolution. As the disease advances, epithelial differentiation is disrupted and a partial EMT emerges, mediated by transcription factors such as SNAIL, TWIST, and ZEB, which promotes cell migration and invasive growth. Inflammatory mediators (e.g., IL-1, IL-6, TNF-α, COX-2) and MMPs alter the tumor microenvironment and promote matrix degradation, angiogenesis, and invasion. The progression of AK to invasive cSCC is based on the interplay of these genetic, epigenetic, and microenvironmental changes.

Despite substantial clinical and experimental evidence linking numerous molecular alterations to the progression of AK, none of the biomarkers discussed in the preceding sections have been clinically validated as a single predictor of malignant transformation in individual AK lesions. This is primarily attributable to the pronounced molecular heterogeneity among lesions, the lack of standardized diagnostic cut-off values, and the limited sensitivity and specificity of individual markers. No distinct factor is consistently altered in all progressing lesions, and many are also detected in non-progressive AKs or in non-neoplastic conditions, including chronic UV exposure, wound healing, and cutaneous inflammation, thereby substantially limiting their specificity.

**Figure 1:**
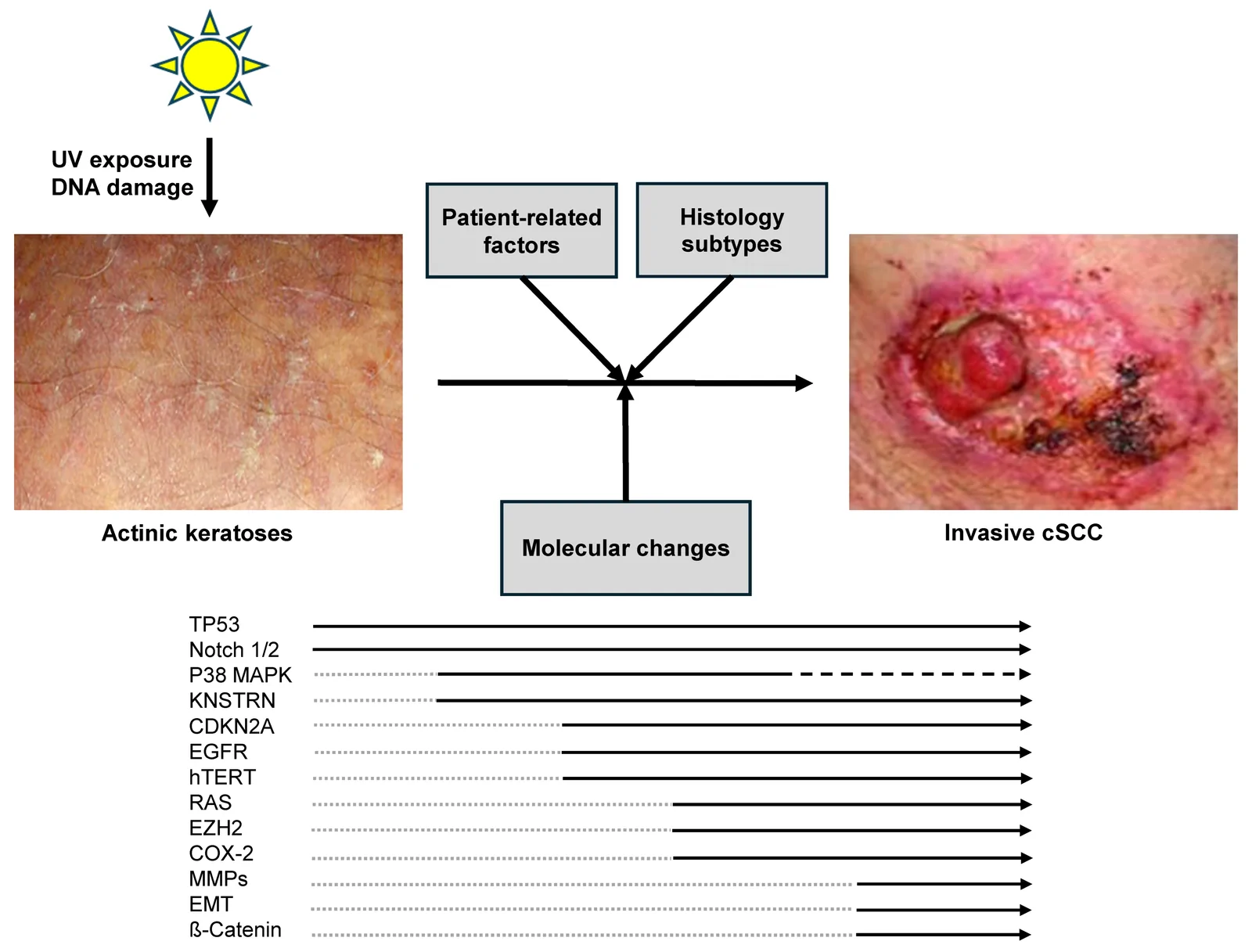
Factors associated with malignant progression of AK. Patient-related factors are detailed in Table 1. Histological subtypes are described in Chapter 4. Arrows next to the molecular factors indicate the temporal pattern of molecular dysregulation. The molecular evolution from AK to invasive cSCC is characterized by the accumulation of partially overlapping genetic, epigenetic and signaling alterations rather than by a uniform linear sequence of molecular events. The available evidence supports a temporal enrichment of distinct molecular alterations across the AK–cSCC continuum, with early alterations affecting genomic integrity and keratinocyte differentiation (*TP53*, *NOTCH1/2*, p38 MAPK, *KNSTRN*), followed by progressive dysregulation of cell-cycle, proliferative and epigenetic pathways (*CDKN2A*, EGFR, RAS, hTERT, EZH2, COX-2) and, at later stages, mechanisms involved in extracellular matrix remodeling, altered cell adhesion and invasion (β-catenin, MMPs, EMT-related factors).

Current evidence suggests that the progression of AK to invasive cSCC is driven by the cumulative dysregulation of multiple, individually variable molecular pathways rather than by alterations in a single signaling cascade (Figure 1). Consequently, the greatest clinical promise lies in integrated molecular signatures that combine genetic, epigenetic, proliferative, inflammatory, and microenvironmental biomarkers. However, which biomarkers should be included in such a panel and how they should be weighted remains largely unresolved. Importantly, recent high-throughput omics studies, including single-cell RNA sequencing, transcriptomic profiling, microRNA analyses, and metabolomic approaches, as well as bioinformatic analyses using gene expression databases, have identified numerous additional genes, miRNAs, and metabolites that are differentially expressed during malignant progression [145,146,147,148]. In principle, all these candidates may represent potential biomarkers of AK progression. As part of establishing them as progression marker, it is necessary to define standardized threshold values and to evaluate their ability to predict malignant transformation in appropriately designed clinical studies that should fulfill several key requirements:

- a prospective longitudinal design with lesion-level follow-up;
- clearly defined and clinically relevant clinical and histopathological endpoints for malignant transformation;
- standardized sampling and, whenever feasible, longitudinal assessment of the same lesion over time;
- evaluation of candidate biomarkers in sufficiently large, well-characterized, and independent patient cohorts;
- systematic assessment of diagnostic and predictive performance, including sensitivity, specificity, positive predictive value, and negative predictive value;
- external validation in independent patient populations; and
- direct comparison of individual biomarkers with multimarker models integrating complementary molecular pathways and relevant clinical and histopathological characteristics.

Nevertheless, prospective longitudinal studies evaluating their ability to predict malignant transformation are still lacking.

Beyond molecular alterations, several patient-specific, clinical, and histopathological characteristics, including advanced age, skin phototype, lesion burden, histological subtype and basal proliferation pattern, have also been associated with an increased risk of progression to invasive cSCC. However, none of these parameters alone provides sufficient predictive accuracy for clinical decision-making. Therefore, the most promising strategy for identifying AK lesions at highest risk of malignant transformation will likely be the development of a multimodal risk model. Such a model could incorporate complementary molecular pathways and clinical or histopathological characteristics, with individual factors weighted according to the strength of their association with progression. Prospective validation will ultimately be required to determine whether this approach can improve risk stratification beyond individual biomarkers.

## Figures and Tables

**Table 1:** Patient-related risk factors of AK progression.

Risk Factor	Mechanism
Chronic UV exposure (occupational and leisure-related)	Long-term cumulative light damage, UV-induced mutations (e.g., *TP53*, *NOTCH*, *CDKN2A*)
Advanced age	Cumulative UV exposure and reduced DNA repair
Light skin type (Fitzpatrick I–II)	Lower natural UV protection
Immunosuppression	Reduced immune surveillance, particularly following organ transplantation with long-term immunosuppressive therapy
History of cSCC	reflects extensive field cancerization, high cumulative UV-induced mutational burden, and increased propensity for clonal evolution within actinically damaged skin
Multiple AK lesions	Expression of chronic UV damage, field cancerization
Male gender	Greater cumulative UV exposure, occupational risk factors
Genetic predisposition	For instance, DNA repair defects as in Xeroderma pigmentosum
HPV infection	Some HPV types can promote carcinogenesis, particularly relevant in cases of immunosuppression
Smoking	Oxidative stress and exposure to carcinogenic substances

**Table 2:** Molecular markers of the progression from AK to cSCC.

Marker	Function	Change During Progression	Remarks
*TP53* (p53)	Tumor suppressor, cell cycle control	Mutation and overexpression	Early mutations in AK (driver mutations); increasingly mutated in cSCC.
NOTCH1/2	Keratinocyte differentiation, tumor suppression	Loss-of-function-mutation	Frequent in cSCC; sometimes in advanced AK
*CDKN2A* (p16INK4a)	Cell-cycle inhibition	Overexpression, mutation, LOH, promoter-methylation	Overexpression in AK; deletions, mutations, or epigenetic alterations more frequent in cSCC
EGFR	Cell proliferation, survival	Overexpression	Increasing expression from AK to cSCC
MMPs (1,2,9,13)	Matrix degradation, invasion	Increased expression	Correlates with invasive potential of tumor cells
EMT factors	Cellular motility and invasiveness	Loss of epithelial cell adhesion, and gain of mesenchymal characteristics	Contributes to epithelial remodeling and promotes early stages of cutaneous carcinogenesis
EZH2	epigenetic regulation of differentiation-associated and tumor suppressor genes	Increased expression	Expression increases steadily from normal skin to AK and invasive cSCC
COX-2	Inflammation, proliferation, angiogenesis	Overexpression	Increased in AK, but even more pronounced in cSCC
*hTERT*-Promoter	Telomere maintenance immortality of UV-damaged keratinocytes	Overexpression and mutation	Telomerase activity detectable in AK but higher in cSCC. Mutations are more frequent in cSCC than in AK
Ras	Activation of MAPK/ERK pathway to promote cell proliferation	Functional activation, activating mutations	Functional activation is more important in AK progression as classic RAS mutations are rare in AK and cSCC
SFKs, Srcasm	Regulation of proliferative and invasive signaling pathways	increased activity (SFKs); reduced expression (Srcasm)	SFK-activation mainly results from EGFR overexpression or the loss of negative regulators
KNSTRN	Chromosome alignment and segregation during cell division	Mutation	Mutations more frequent in AKs with PRO II/III and invasive cSCCs than in healthy skin or AKs with PRO I
β-Catenin	Regulation of cell proliferation and differentiation through Wnt-signaling pathway	Shift from membranous to cytoplasmic/nuclear localization	increased cytoplasmic and nuclear localization during progression from AK to cSCC
DNA-methylation	Regulation of gene expression	Increased promoter-methylation	*CDKN2A* and E-Cadherin promoter methylation is associated with AK progression
miRNAs	Regulation of gene expression	Increased expression of oncogenic miRNAs and reduced expression of tumor suppressor miRNAs	Oncomir-1 cluster, miR21 and miR31 are increased in cSCC, whereas miRNA-34a and miR 203 are downregulated

## Data Availability

No new data were created or analyzed in this study. Data sharing is not applicable to this article.
